# Description of Novel Molecular Factors in Lumbar DRGs and Spinal Cord Factors Underlying Development of Neuropathic Pain Component in the Animal Model of Osteoarthritis

**DOI:** 10.1007/s12035-023-03619-x

**Published:** 2023-09-21

**Authors:** Natalia Malek, Jakub Mlost, Magdalena Kostrzewa, Jolanta Rajca, Katarzyna Starowicz

**Affiliations:** 1 Department of Neurochemistry, Maj Institute of Pharmacology, Polish Academy of Sciences, Krakow, Poland; 2 Department of Chemical Biology and Bioimaging, Faculty of Chemistry, Wroclaw University of Science and Technology, Wroclaw, Poland; 3 Galen Orthopaedics, Bierun, Poland; 4 Galen Lab, Bierun, Poland

**Keywords:** Osteoarthritis pain, Neuropathic pain component, Pain chronicity factors expression, MIA model, Behavioral studies, Knee microtomography

## Abstract

Osteoarthritis (OA) is one of the most common joint disorder, with pain accompanied by functional impairment, as the most pronounced clinical symptom. Currently used pharmacotherapy involves symptomatic treatment that do not always provide adequate pain relief. This may be due to concomitance of central sensitization and development of neuropathic features in OA patients. Here we performed studies in the animal model of OA to investigate of the neuropathic component. Intraarticular injection of monoiodoacetate (MIA, 1 mg) was used to induce OA in Wistar male rats. Development of pain phenotype was assessed by behavioral testing (PAM test and von Frey’s test), while corresponding changes in dorsal root ganglia (DRGs L3–L5) and spinal cord (SC) gene expression were assessed by means of qRT-PCR technique. We also performed microtomography of OA-affected knee joints to correlate the level of bone degradation with observed behavioral and molecular changes. We observed gradually developing remote allodynia after MIA treatment, indicating the presence of neuropathic component. Our results showed that, among DRGs innervating knee joint, development of central sensitization is most likely due to peripheral input of stimuli through DRG L5. In SC, development of secondary hypersensitivity correlated with increased expression of TAC1 and NPY. Our studies provided molecular records on abnormal activation of pain transmission markers in DRG and SC during development of OA that are responsible for the manifestation of neuropathic features. The obtained results increase insight into molecular changes occurring in the neuronal tissue during OA development and may contribute to readdressing treatment paradigms.

**Supplementary Information:**

The online version contains supplementary material available at 10.1007/s12035-023-03619-x.

## Introduction

Osteoarthritis (OA) is the world’s leading cause of disability among elderly and it has been recognized by the World Health Organization (WHO) as a “priority disease” (report WHO/EDM/PAR/2004.7) and one of the top 5 healthcare costs in Europe [1]. OA is a degenerative joint disease caused by breakdown of cartilage and underlying subchondral bone, leading to development of chronic pain characterized by neuropathic features such as feelings of burning pain, tingling, numbness [2], and development of hyperalgesia at sites distant from the injury [3]. It is estimated that pain in one-third of OA patients involves neuropathic component [4]. Constant and reoccurring pain at resting state may eventually lead to development of depressive symptoms and sleep disorder [5]. Unfortunately, there is no objective measurement available to predict development of neuropathic component, nor severity of pain, based on bone/cartilage damage or the intensity of synovial inflammation [6].

Importantly, cartilage itself is not innervated. Thus, it is not surprising that up to 40% of individuals with radiographic damage have no pain, while patients with minimal and even non-radiographically detectable cartilage abnormalities develop pronounced, debilitating pain [7]. It is only surrounding tissue such as the synovium, ligaments, and subchondral bone that are largely innervated by a dense network of myelinated and unmyelinated fibers, which are involved in mediating pain sensation induced by joint lesions. Aδ myelinated fibers respond to strong mechanical stimuli and thermal stimuli, whereas unmyelinated C fibers, which are normally inactive, become responsive in pathological conditions such as inflammation to mechanical, thermal, and chemical stimuli [8]. In animal model of OA, the firing rate of C and Aδ afferents was increased after intra-articular (i.a.) MIA injection [9], whereas in the surgical model of OA, increased numbers of dorsal root ganglion (DRG) neurons responded to physical stimuli directed toward the operated knee or ipsilateral hind paw, compared to sham-operated mice. This was previously shown to be correlated with the presence of knee hyperalgesia and mechanical allodynia [10]. In OA, local inflammation is an important part of pathophysiology of the generation and maintenance of joint pain and it is accompanied by release of proinflammatory factors, implicating their role in nerve sensitization. Indeed, recent findings correlate inflammation of the synovium with development of knee pain sensitization [11]. Nonetheless, very few studies on the mechanisms underlying occurrence of the neuropathic pain component in OA development are available. Local release of proinflammatory factors leads to sensitization of nerve endings, while further progression of the neuronal input is largely unknown [12].

In OA and other chronic pain conditions, there is a growing number of reports that central mechanisms and sensitization play a significant role. In fact, central mechanisms seem to be engaged mostly during late and chronic stages [13] and may be responsible for the failure of treatments aiming at inflammatory component of the pain [14]. The correlation of OA development with signs of neuropathy was shown previously by Thakur et al., although this report focused on changes of histopathological and immunofluorescence features rather than quantitative alterations of gene expression [15]. The latter would be important to estimate to distinguish underlying mechanisms leading to development of neuropathic component. Moreover, interplay between the central and peripheral systems suggests a general plasticity of the nociceptive system in OA pain [16] that requires further studies.

Therefore, in our studies, we focused on establishing molecular factors involved in development of peripheral and central sensitization in DRGs and spinal cord (SC) in the MIA model of OA. We performed behavioral evaluation of pain symptoms development measuring response to direct mechanical stimulus (pressure application measurement, PAM) and presence of tactile allodynia (von Frey’s test). As our behavioral results suggested development of neuropathic component in our experimental setup, we have associated development of direct and distant hypersensitivity in OA with changes in subchondral bone morphology after MIA injection in time. The results showed possibility of exposure of nerve endings; thus, we examined increased proinflammatory signaling in DRGs that are innervating knee joints. Furthermore, we performed analysis of gene expression that are known to be affected during development of neuropathic pain, in mentioned DRGs and SC tissue.

## Methods

### Animals

Male Wistar rats (Charles River, Hamburg, Germany) initially weighing between 225 and 250 g were used for all the experiments. The authors are aware of possibility of biased results due to sex limitation in the study; nevertheless, to obtain reliable data in relatively small group of animals, they decided to perform experiments in males exclusively. The rats were housed in groups of 5 animals per cage under a 12:12-h light/dark cycle and had free access to food and water. All animals were allowed to acclimatize to their holding cages for 3 to 4 days before any behavioral or surgical procedures were conducted. In total, 100 animals were used in performed experiments (20 animals in phenotype development and immunofluorescence studies; 40 animals in pharmacological studies and 40 in microtomography and molecular studies). Animals were allocated in experimental groups randomly. All the behavioral experiments were conducted between 9:00 AM and 12:00 PM. The experiments were performed following the guidelines of the IASP and with the approval number 1130/2014 of the Local Bioethics Committee of the Institute of Pharmacology (Krakow, Poland). Care was taken to implement the “3 Rs” rule (replacement, reduction, and refinement) to reduce the number of animals used and their suffering during the experiments.

### OA Induction

Animals were deeply anaesthetized with 5% isoflurane in 100% oxygen (4.5 L/min) until the flexor withdrawal reflex was abolished. The skin overlying the right knee joint was shaved and swabbed with 100% ethanol. A 27-gauge needle was introduced into the joint cavity through the patellar ligament and 1 mg of MIA (sodium monoiodoacetate, Sigma-Aldrich), which is an irreversible NADPH inhibitor, diluted in 50 µL 0.9% saline was injected into the joint (intra-articular, i.a.) to induce OA-like lesions. MIA inhibits chondrocyte glycolysis and produces cartilage degeneration and subchondral bone alterations. The MIA model reproduces osteoarthritis-like histological lesions and functional impairment like that observed in human disease [17]. In biochemical experiments, sham animals (after intraarticular injection of saline) were used as a control. Animals were allocated to groups randomly. The rats were sacrificed at day 28 after MIA injection.

### Pressure Application Measurement

The pressure application measurement (PAM) device (PAM; Ugo Basile, Italy) has been used for the mechanical stimulation and assessment of joint pain as described previously [18]. Briefly, a quantifiable force was applied directly to the affected knee joint, and the automatic readout of the response was recorded. The animals were held lightly, and the operator placed a thumb with a force transducer mounted unit on one side of the animal’s knee joint and a forefinger on the other. A gradually increasing squeeze force was applied across the joint at a rate of approximately 30 g/s with a maximum test duration of 15 s or applied 500 g force. Using calibrated instrumentation, the force in grams applied was displayed on a digital screen and was recorded. The test end point was the point at which the animal withdrew its limb or showed any behavioral signs of discomfort or distress such as freezing of whisker movement, wriggling, or vocalizing, such as freezing of whisker movement or wriggling. Two measurements of the ipsilateral knee were obtained and the mean limb withdrawal threshold (LWT) in grams (gf) was recorded. The baseline measurements were obtained immediately before the intra-articular injection (postoperative day 0) and then on 2, 7, 10, 16, 21, and 28 postoperative days. The experimenters were blinded to the experimental groups throughout measurements.

### von Frey’s Test

Touch-evoked pain is a hallmark of neuropathic pain both in animal models and in pain patients [19]; therefore, development of mechanical allodynia was assessed in our studies. For the assessment of mechanical allodynia, the von Frey test was used. Animals were tested for their paw withdrawal threshold in response to the automatic von Frey’s filament (Bioseb, France). Rats were placed in plastic cages with wire net floor 15 min before the experiment (additional acclimatization was performed for 3 days before behavioral experiments started). A von Frey filament was applied to the midplantar surface of the ipsilateral hind paw and limb withdrawal threshold in grams (g) was recorded. Measurements were taken twice and average of LWT values was drawn. The baseline measurements were obtained immediately before the intra-articular injection (postoperative day 0) and then on 2, 7, 10, 16, 21, and 28 postoperative days. The experimenters were blinded to the experimental groups throughout measurements.

### TaqMan Quantitative Real-Time Polymerase Chain Reaction

Rats were sacrificed by decapitation. DRGs (L3–L5) and dorsal horn of the SC were collected for each animal both on ipsilateral and contralateral side. Tissue samples were placed in individual tubes with the tissue storage reagent RNAlater (Qiagen, Inc.), frozen on dry ice, and stored at − 80 °C until RNA isolation. The samples were homogenized in 1 mL of Trizol reagent (Invitrogen, Carlsbad, CA). The RNA isolation was performed according to the manufacturer’s protocol. The total RNA quantity was assessed using a Nanodrop spectrophotometer (ND-1000, Nanodrop; Labtech International, UK). Each sample was equalized to a concentration of 1 μg/μL and reverse transcribed to cDNA using iScript Reverse Transcription Supermix (BioRad, Hercules, CA, USA) according to the manufacturer’s protocol in a 20 µL total volume. The qPCR reactions were performed using Assay-On-Demand TaqMan (Applied Biosystems). The following assays were performed: Rn02531967_s1 (*Bdnf*), Rn00580432_m1 (*Il1b*), Rn01410330_m1 (*Il6*), Rn01533872_m1 (*Ngf*), Rn01410145_m1 (*Npy*), Rn01500392_m1 (*Tac1*), Rn01525859_g1 (*Tnf*), Rn00569199_m1 (*Cgrp*), Rn01527840_m1 (*Hprt1*), Rn00591020_m1 (*Scn9a*), and Rn01485332_m1 (*Scn3a*). The reactions were run on a Real-Time PCR CFX96 Touch System (Bio-Rad). The expression of the *Hprt1* transcript with a stable level between the control and investigated groups was quantified to control for variation in the cDNA amounts. The threshold cycle (CT) value for each gene was normalized with the CT value of *Hprt1*. RNA abundance was calculated as 2^− (normalized ΔCt)^. Experiments were run in triplicates and mean value was calculated. The results are presented as a fold change proportional to the expression level in sham animals.

### Immunohistochemistry

The rats were anesthetized with sodium pentobarbital (60 mg/kg) and perfused through the ascending aorta with 100 mL of 0.9% saline followed by 300 mL of 4% paraformaldehyde in 0.1 M phosphate buffer (PB), pH 7.4. The lumbar DRGs were located by tracing the lumbar dorsal roots back to the sciatic nerve. The dissected tissue was postfixed for 2 h at 4 °C, cryoprotected in 30% sucrose in 0.1 M PB for 12 h at 4 °C, and embedded in Tissue Tek (OCT; Miles, Inc., Elkhart, IN). Cryosections were cut and thaw mounted onto Superfrost slides (Menzel, Germany) at a thickness of 12 µm. The sections were processed for immunohistochemistry. The slides were incubated with blocking solution (TBS containing 10% normal goat serum and 0.1% Triton) for 2 h at room temperature. A rabbit primary antibody against rat IL1β (Abcam, ab9787) and CGRP (Sigma Aldrich, C8198) proteins were diluted in blocking solution at 1:50 and 1:200 ratio, respectively. Slides were incubated overnight with primary antibody. After 3 washes in PB, the primary immunofluorescence was revealed by incubation for 2 h in a mixture of goat anti-mouse antibody conjugated with Alexa 555, diluted to 1:500 in blocking solution. The sections were examined, and the areas of interest were photo documented on a confocal laser scanning microscope, DMRXA2 TCS SP2 (Leica Microsystems, Germany), with a × 20 dry objective lens (Leica) driven by confocal software (Leica), Gre/Ne laser (laser line emitted at 543 nm light); the background noise of each confocal image was reduced by averaging 8 scans/line and 8 frames/image. Compared slides were stained simultaneously and the images were collected with the same exposure settings to minimize variances in staining intensity between the slides. Cells expressing IHC signal intensity above 66% of image histogram values were considered positive. Images were analyzed using automated pipeline (raw images were first normalized to extend the histogram to values between 0 and 255. Then, images were binarized and to count only positive cells, we have applied threshold value above > 170. Further image processing included removal of outliers, dilution, and watershed. Finally, particles with size above 80 px and circularity between 0.35 and 1 were counted as cells; Supplemental Fig. 1) and then validated by blinded observer.

### X-ray Microcomputed Tomography (XMT)

**Fig. 1:**
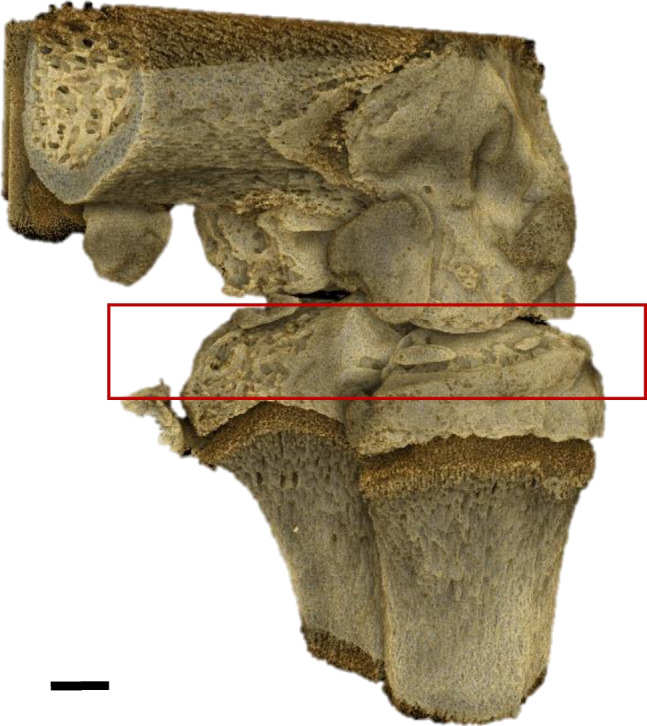
3D visualization of the femorotibial joint. The analyzed volume of subchondral bone of tibia was marked. Scale bar = 1 mm

The ex vivo commercial XMT system was used (v|tome|x s, GESensing & Inspection Technologies, Phoenix|x-ray, Wunstorf, Germany). Trimmed knee samples with tibial and femoral bone sections were dissected before the intra-articular injection of MIA (postoperative day 0) and then on 10, 21, and 28 post MIA injection and immediately stored in dry ice (− 80 °C) until analysis. The samples were defrosted for 30 min prior to analysis. The XMT scanning parameters were as follows: System Phoenix v|tome|x s; voltage (kV): 160; current (μA): 70; voxel size (μm): 11.3; detector timing (milliseconds): 200; filter Cu: 0.1. Identical scanning parameters were applied to all the samples. Each sample was placed inside the scanner chamber using the same holder. Reconstructed cross-sections were stored in 256 grayscale format (8 bits per voxel) and later processed by Drishti (open-source Volume Exploration and Presentation Tool by Limaye) [20] to visualize the bone microstructure. All advanced image analyses were carried out with the ImageJ software, an open-source image enumeration software package (US National Institute of Health, Bethesda, MD, USA) [21] and CTAn (Skyscan CT-analyzer software, Belgium). The microstructural parameters were estimated for subchondral bone of tibia (Fig. 1), including the following: bone volume (BV)—volume of the region segmented as bone; specific bone surface (BS/BV)—the ratio of the segmented bone surface to the segmented bone volume; the trabecular thickness (Tb.Th)—the average thickness of trabeculae; total porosity (Po(tot))—the volume of all the open and closed pores as a percentage of the total volume [22].

### Data Analysis

The analysis was performed using Prism 7 (GraphPad Software). Data was first examined for Gaussian distribution by Shapiro–Wilk normality test and the equality of variances by Brown–Forsythe or *F* test. Behavioral data regarding pain phenotype development was analyzed using one-way ANOVA with Dunnett’s post hoc test, where mean values at each time point (day post MIA injections) were compared against basal mean values at day 0. Changes in expression patterns were analyzed by one-way ANOVA with Bonferroni’s post hoc test, where mean values were compared against samples from sham-treated animals. Quantitative IHC results were compared in each DRG using two-tailed unpaired *t*-test. In the case of the XMT results, the data did not meet the assumptions about Gaussian distribution and equality of variances in Tb.Th; therefore, it was analyzed by non-parametric Kruskal–Wallis test with Dunn’s post hoc comparison, whereas BS/BV and total porosity were analyzed by ANOVA with Dunnett’s post hoc test, where mean values at each time point (day post MIA injections) were compared against basal mean values at day 0. XMT data underwent an outlier analysis with ROUT method and *Q* value set to 5%. All experiments were performed for *n* = 6–10. For qRT-PCR analysis, samples of DRGs from two animals (separately for ipsilateral and contralateral L3, L4, L5) were pulled together to obtain better RNA yield. Behavioral analyses were performed under blinded conditions.

## Results

### Development of Pain Phenotype and Disturbances in Subchondral Bone Morphology After Intraarticular Administration of MIA

**Fig. 2:**
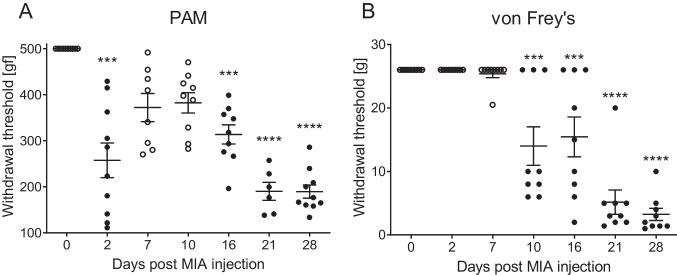
Development of pain phenotype after intraarticular administration of MIA (1 mg) measured in PAM (**A**) and von Frey’s (**B**) tests. A statistical analysis was performed using a one-way ANOVA followed by Dunnett’s post hoc test. The values are presented as mean ± SEM; *n* = 6–10. *Statistically significant difference (*P* < 0.05)

**Fig. 3:**
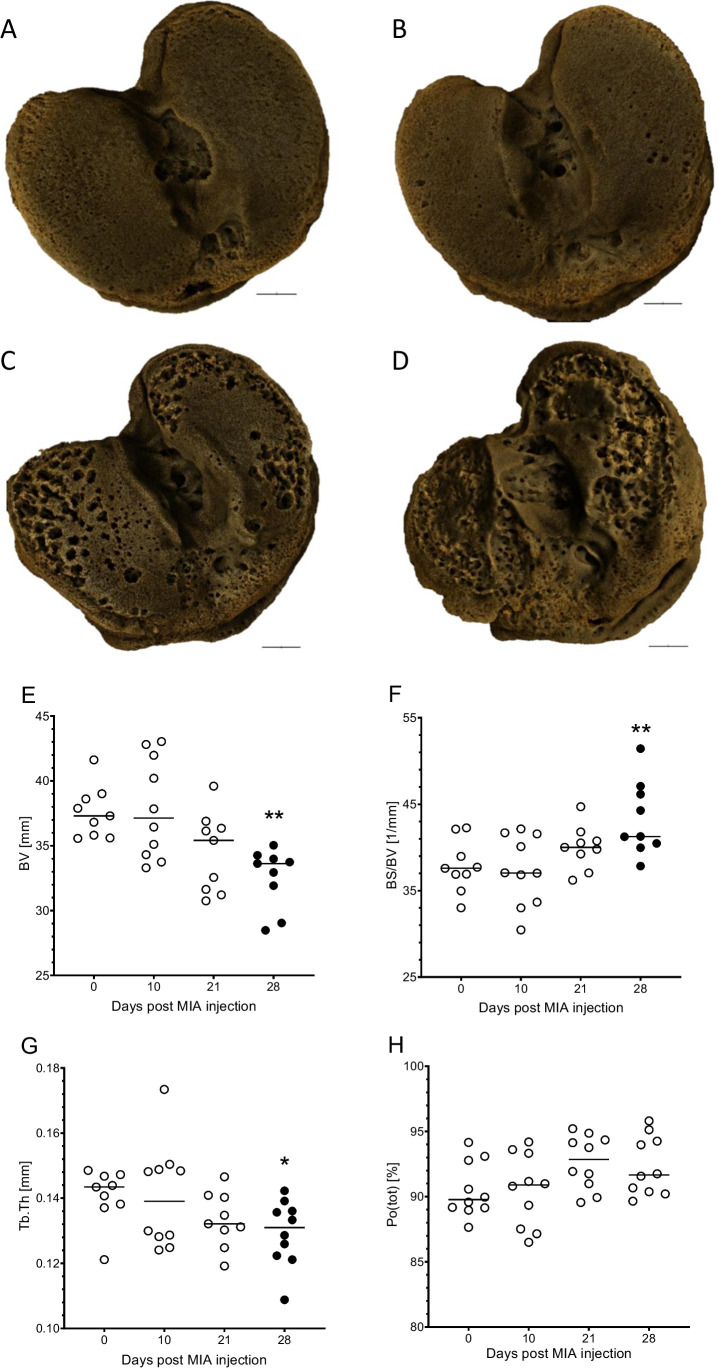
Development of histomorphological changes due to MIA administration in microtomographic image of the subchondral bone. 3D visualizations present articular surface of the tibia at day 0 (**A**), day 10 (**B**), day 21 (**C**), and day 28 (**D**) after OA induction. Black scale bar represents 1 mm. Graphs present quantitative analysis of subchondral bone morphology including segmented bone volume (**E**), ratio of the segmented bone surface to the segmented bone volume (**F**), mean thickness of trabeculae (**G**), and total porosity (**H**). Each experimental group included *n* = 8–10 rats, as outliers were detected by ROUT method excluded from analysis. Statistical analysis was performed using one-way ANOVA followed by Dunnett’s post hoc test or Kruskal–Wallis test followed by Dunn’s post hoc test, both with *P* < 0.05 confidence interval. Filled black circles denotes statistical significance in comparison to day 0

Intraarticular injection of MIA caused significant reduction in mechanical pain threshold, measured in PAM test at post-operative day 2 and further from day 16 till the end of experiment at day 28 (Fig. 2A). Allodynia measured as withdrawal threshold by von Frey’s test was observed from day 10 and increased gradually until day 28 (Fig. 2B) indicating development of neuropathic component in OA pain over time. Moreover, it has to be stressed that only 28 days post MIA injection all animals present significant allodynia that may be due to the different pace of cartilage degradation for different animals. Further biochemical analyses were performed starting from day 10, although analyses of neuropathic factors’ expression were performed at day 28. To back up our hypothesis, we performed XMT analysis of tibial articular surface in rats after MIA administration (Fig. 3A–D). Quantitative analysis of subchondral bone morphology revealed statistically significant decrease in BV and Tb.Th (Fig. 3E, G), as well as increase in BS/BV (Fig. 3F) at day 28 after OA induction. Albeit we have not observed statistically significant changes in total bone porosity (Po(tot)) in any given time point (Fig. 3F), a *t*-test comparison between D0 and D28 revealed *P* = 0.0773, which suggests a raising trend in bone porosity due to MIA administration.

### Immunohistochemical Analysis of Number of Cells Positive for IL1β and CGRP in Lumbar DRGs of Animals Treated with MIA

**Fig. 4:**
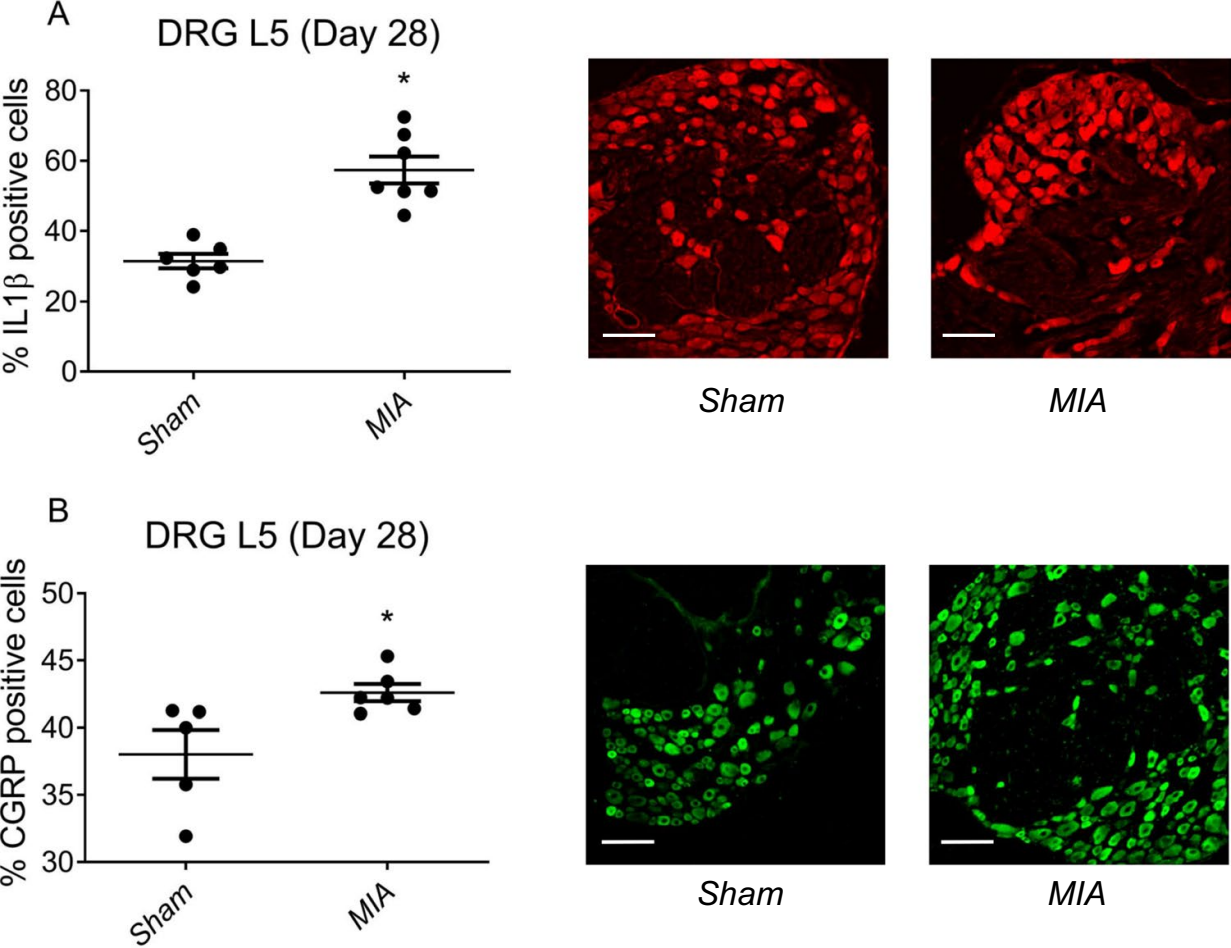
Changes in the expression of IL1β (**A**) or CGRP (**B**) in DRG L5 after intraarticular injection of MIA (1 mg). Data is presented as % of positive cells ± SEM. Experiments were performed double-blinded. The results were evaluated using a *t*-test analysis; *n* = 10–12 slices. *Statistically significant difference (*P* < 0.05). White scale bar denotes 100 µM

The increased bone porosity leads to the exposure of nerve endings at the final stages of the experiment and in consequence to activation of neuropathic ascending pathways; therefore, we decided to examine factors of neuronal origin that are known to be activated DRG during development of neuropathic pain—IL1β and CGRP [23]. Immunohistochemistry analysis revealed IL1β immunofluorescence at day 28 in DRG L5 in both sham- and MIA-treated animals, although the number of IL1β cells was higher in animals with developed OA (Fig. 4A). Similar situation was observed for CGRP immunofluorescence that was detected in higher number of DRG L5 cells in MIA-treated animals when compared to control (Fig. 4B). The number of positive cells both for IL1β and CGRP in DRG L3 was similar for sham- and MIA-treated animals, while in DRG L4 was significantly increased (Supplemental Fig. 2). Nonetheless, the enhancement was lower than for DRG L5 suggesting that neuronal input in that model goes mainly through the latter.

### Changes in Expression Patterns of Genes Involved in Inflammation and Pain Processing in DRG and SC During OA Development

**Fig. 5:**
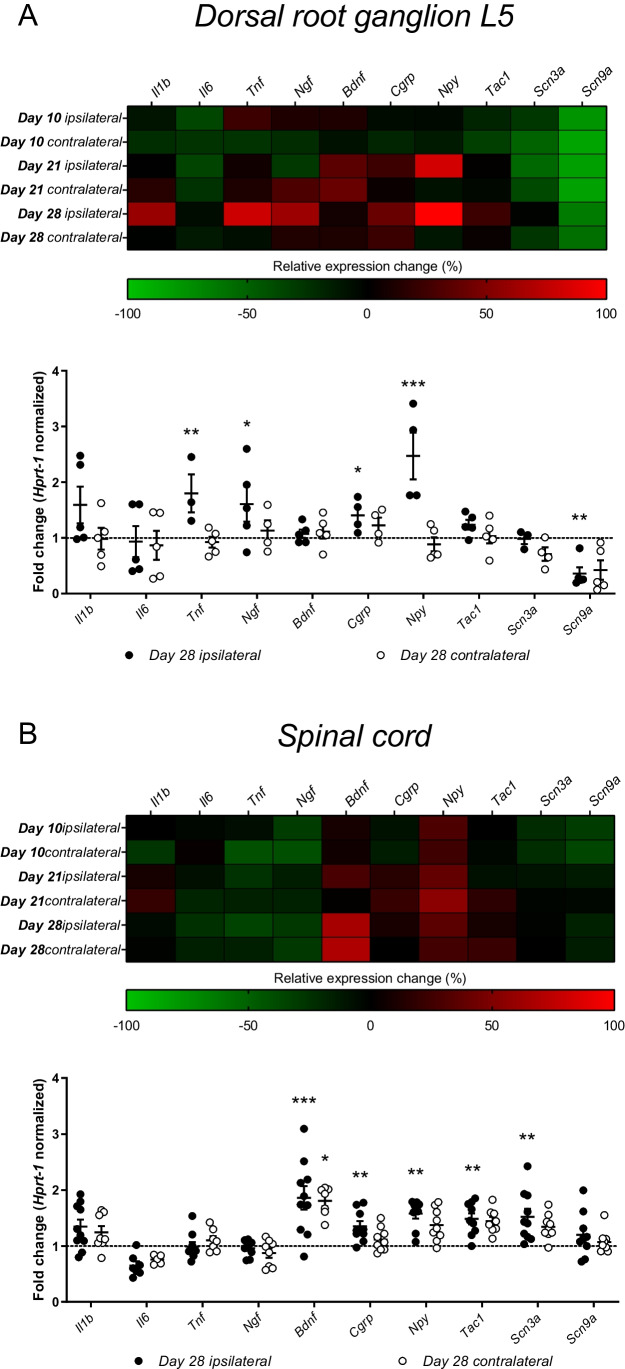
Changes in the neuropathic pain-related gene expression in the DRG L5 (**A**) and lumbar SC (**B**) during development of OA after intraarticular injection of MIA (1 mg). Data are presented as the mean ± SEM and represent normalized averages derived from 6–10 samples for each group. The results are presented as a fold change normalized to the expression of the reference gene *Hprt1* compared to the sham animals. Statistical analysis was performed using one-way ANOVA followed by Bonferroni post hoc tests. Values with *P* < 0.05 were considered significant. *Significant differences vs. sham

Our results showed that, among lumbar DRGs innervating knee joint—L3, L4, and L5, OA development was mostly affecting gene expression in DRG L5 (data for DRG L3–L4 can be found in Supplemental Fig. 3). Twenty-eight days post MIA injection, we observed most abundant changes in chosen factors’ expression in both DRG L5 and lumbar SC (Fig. 5). Among inflammatory markers, mRNA for Il1β (*Il1b*) tends to be increased in both neuronal structures, though *Il6* transcript showed no level alterations. Significant increase of *Tnf* and *Ngf* was observed in DRG L5, while *Bdnf* expression was upregulated at both ipsilateral and contralateral SC at the given time point. Further pro-nociceptive neuropeptide examination showed increase in the *Cgrp* and *Npy* expression in both studied structures. The level of the transcript for preprotachykinin-1 (*Tac1*)—a precursor for neurokinin A and substance P—was significantly elevated in SC, suggesting increased response of CNS to tactile stimuli from peripheral nervous system in the studied model. We also observed changes in expression of voltage-gated sodium channel subunits (NaV1.3 and NaV1.7) in both DRG L5 and SC. Transcript for NaV1.3 (namely *Scn3a*) was overexpressed in lumbar SC after development of OA in animals; on the contrary, expression of *Scn9a* (NaV1.7) was downregulated in DRG L5 in the measured time point.

## Discussion

Experiment performed within our study revealed nuanced pain phenotype that may account for both nociceptive and neuropathic component. First, we have observed a biphasic decrease in withdrawal threshold in PAM measurement, as it was described in details in our previous work [18]. The initial stage of pain is commonly attributed to inflammation resulting from the procedure, while subsequent to pain resolution (as seen at days 7 and 10), we observed a second phase of heightened knee sensitivity attributed to cartilage degradation and the progression of osteoarthritis (OA). Decrease in mechanical pain threshold in PAM precedes development of tactile allodynia (von Frey’s test), which is in line with our further findings showing increase in expression of numerous inflammatory mediators, pro-nociceptive peptides in DRGs, and SC at late stage of OA development. These features of the behavioral phenotype were quite unusual for inflammatory pain, particularly the time course or the site of the allodynia (distinct from injury site). The time course of the behavioral phenotypes were intriguing, particularly when comparing the PAM and von Frey responses of days 2–7 to those of days 21–28. These data support the possibility of a potential neuropathic (i.e., nerve injury) mechanism. Hypersensitivity to mechanical stimuli could be observed as early as day 10 after MIA injection in some of the animals, although full effect was reached at the end of experiment (Fig. 2). Those results are corresponding with microtomography images, where first tissue deficiencies were observed at day 10 after model induction and cartilage continue to decay until termination of the experiment (Fig. 3B). Here for the first time, we were able to demonstrate with the use of microtomography that 1 mg of MIA injection is sufficient to cause cartilage degradation analogical to one observed in clinical conditions and simultaneously lead to development of OA-like hypersensitivity in rats. It is important as the dose 3 mg of MIA used in our lab previously may lead to over excessive changes in the cartilage metabolism, which may not correspond with OA development seen in patients [24].

Microtomographic data led to obtain 3D visualization of knee joints. Based on the visualization of tibial articular surface, its degradation can be precisely observed in specific time points (Fig. 3). The articular degeneration also caused deformity to subchondral bone. These visual observations were confirmed by analyzing microstructural parameters, such as BV, BS/BV, Tb.Th, and total porosity. The results obtained in this study show that OA causes damage of cortical and trabecular bone, such as significant quantitative loss of bone volume (BV), gradual decrease in trabecular thickness (Tb.Th), and increase in BS/BV and total porosity in subchondral bone. BS/BV ratio indicates the level of bone turnover [25], which is accelerated in OA development [25].

The increased bone porosity leads to the exposure of nerve endings at the final stages of the experiment and in consequence to activation of neuropathic ascending pathways. It was shown previously by Thakur et al. that development of hypersensitivity in MIA model of OA was accompanied by the increase of ATF-3 expression in lumbar DRGs [15]. Our studies showed increase expression of *Atf3* (data not shown), although we focused on examination of the neuronal input to the central nervous system (CNS), and observed increase in the number of lumbar DRG cells expressing factors that are known to be activated during development of neuropathic pain—IL1β and CGRP [23, 26]. Moreover, both factors are known to be expressed in high-threshold mechanonociceptive Aδ fibers that are responsible for tactile allodynia [27, 28]. The number of cells expressing both factors was increased in the OA animals at the later stage of disease development only in DRG L5, showing that main neuronal input from knee tissues may go through that particular lumbar DRG (Fig. 4), especially that this is in line with anatomical distribution of nerve afferents from knee subchondral bone [29].

A key part of our research was molecular analysis of the expression of inflammatory mediators, pro-nociceptive peptides, and neuronal receptors in DRGs and lumbar SC to point out the possible mechanisms of the development of neuropathic component in OA. Proinflammatory interleukins are well known to be involved in the development of neuropathic pain [26]. Our data demonstrates that the mRNA expression of IL1β in DRGs of MIA animals was elevated on day 28 (Fig. 5A), which aligns with an increased presence of IL1β-expressing cells in the same DRG L5 samples (Fig. 4A). IL1β is widely recognized for its involvement in the development of neuropathic pain, having demonstrated the ability to induce neuropathic pain in vivo [30]. Moreover, various studies have illuminated its impact on dorsal root ganglion neurons [28, 31]. It is important to note, however, that IL1β has not been previously associated with the development of the neuropathic pain component of OA at the DRG level. Nevertheless, we hold that the presence of this interleukin, closely tied to neuropathic pain, in our research supports our findings and substantiates the conceptual underpinning the study. Despite the well-documented elevation of IL6 in DRGs during neuropathic pain development [32, 33], our study might not capture these changes, probably due to our focus on individual DRGs (L3, L4, and L5 separately). Interestingly, upon examining data from other DRGs (L3 and L4), we do observe an elevated expression of IL6 in DRG L3 during the progression of OA-associated pain (Supplemental Fig. 3). Mechanistically, peripheral and central pain sensitization develop with chronic mechanical and inflammatory stimuli in induced and naturally occurring animal models of OA, which is in part mediated by tumor necrosis factor alpha (TNFα), nerve growth factor (NGF), and brain-derived neurotrophic factor (BDNF) [34]. Our data showed increased expression of TNFα in DRG L5 in the response to development of OA already 10 days after MIA injection followed by increase in *Ngf* levels 28 days post model induction. The findings of the present study suggest that the elevated production of TNFα may have induced elevated production of NGF, which is to activate the regeneration paths of peripheral nervous system [35]. In contrast to the central nervous system, the peripheral nervous system has some regenerative potential that is mediated by neurotrophic factors like NGF or BDNF. However, actual regeneration is far from complete and functional recovery rarely returns to pre-injury levels. Moreover, excessive release of neurotrophic factors may backfire into development of malfunctioning neuronal projection, resulting in chronic pain development [35]. Functionally, neuronal somas in DRGs often develop lowered firing thresholds or persistent spontaneous firing following injury, which is assumed to intensify peripheral signals reaching the SC and, thus, may contribute to the phenomenon of sensitization and neuropathic pain [36]. TrkA, TrkB, and TrkC receptors activated by NGF seem to mediate increase of BDNF synthesis in primary afferents [37–39] resulting in hypersensitivity. In the case of the spinal cord, our studies showed increase in *Bdnf* levels starting 21 days post model induction. Increased expression of BDNF is involved in facilitating plasticity of spinal neurons in response to pain input leading to antinociception, although chronic pain can alter the response to elevated BDNF levels allowing the development of central sensitization and contribute to the maintenance of hyperalgesia [40, 41].

The peptidergic factors (CGRP, NPY, and preprotachykinin-1), that expression was upregulated in both SC and DRG in our study, are involved in pain signaling within nervous system. Characteristics of peptides, such as requirement of relatively high threshold value for release, diffused and prolonged action within extracellular space, high binding affinity, and regulation on transcription factors [42], predispose them as physiologically functional modulators in chronic pain. CGRP is released in spinal cord in response to acute noxious stimuli [43]. The primary signal transduction mechanism of CGRP receptors in the spinal cord is activation of adenylyl cyclase [42]. Up to date, acute and persistent increase in CGRP expression in DRG was shown in inflammatory model of arthritis [44]. Moreover, Schaible et al. have shown CGRP release in the dorsal horns of SC due to mechanical innocuous pressure in animals with joint inflammation, while the same stimuli did not evoke any effects in control animals [43]. Indeed, studies of other authors have shown hyperalgesia to mechanical stimuli following acute intrathecal CGRP administration [45, 46]. Likewise, a CGRP receptor antagonist blocks the increased synaptic current evoked in spinal dorsal horn neurons in vitro in samples obtained from rats with acutely inflamed joints [47]. Similarly, intrathecal administration of a CGRP receptor antagonist blocks mechanically evoked nociceptive responses in animals after the development of hyperalgesia due to joint inflammation [48]. Human studies seem to confirm essential role of CGRP in chronic pain development as CGRP is upregulated in synovial fluid and serum of OA patients [49], while the cerebrospinal concentration of CGRP is downregulated [50]. Moreover, the CGRP levels in serum are positively correlated with pain intensity [49]. Our model of OA seems to mimic increase in CGRP progression observed in clinical conditions in both OA and neuropathic pain. NPY receptors are G_i/o_ coupled, resulting mostly in hyperpolarization of neuron upon activation. Therefore, NPY upregulation in DRG and SC observed in models of neuropathic pain [51–54], as well as in our OA model, can be explained as an adaptive inhibitory mechanism to counteract sensitized pain pathways. This hypothesis is strongly supported by conditioned knock-out experiments, in which NPY deletion following nerve injury led to rapid increase in hypersensitivity, indicating tonic inhibitory role of NPY in the maintenance of persistent pain [55]. *Tac1* gene codes for preprotachykinin-1, a precursor protein which can variably undergo post-translational modification to produce neurokinin A and substance P. Recent findings have presented essential role of *Tac1* in the pathophysiology of chronic pain as Tac1 KO prevents development of mechanical sensitization [56]. Apart from inflammation and pain, both neurokinin A and substance P are mediators of nervous tissue damage; therefore, increased levels of those peptides in our model may account for observed development of neuropathic component of pain [57]. Consistence of expression pattern of the studied peptidergic factors in our data (meaning increased expression of most of them 21 and 28 days post MIA injection on the ipsilateral site of injury) points them as probable factors leading to development of neuropathic component in MIA model of knee osteoarthritis. Simultaneously, these results indicate the neural origin of the neuropathic component of OA-related pain, thus diminishing the involvement of inflammation in the development of this phenotype.

Moreover, observed behavioral and neurochemical disturbances in the MIA model of OA were accompanied by changes in expression of sodium channels, a key mediator in signal propagation throughout nervous tissue, including pain sensation. Ectopic spontaneous activity in primary afferent neurons following nerve injury is matched by altered expression of voltage-gated sodium channels [58]. Nav1.7 is preferentially expressed at high levels in nociceptive and sympathetic neurons and its gain of function mutation lowers thresholds for single action potentials and high frequency firing in dorsal root ganglion neurons resulting in inherited pain disorder [59]. In our study, we have observed a downregulation of gene coding for Nav1.7 (*Scn9a*) in DRG L5, which may be a homeostatic response for ongoing inflammation. This also confirms the activation of molecular pathways leading to the development of neuropathic pain, as it was shown that Nav1.7 is colocalizing with CGRP and its firing is responsible for the transduction of pathological pain firing [60]. On top of that, this study showed that overexpression of *Scn9a* was present only in unaffected DRG neurons, which may explain why we observed global downregulation of this transcript in our experiments. It was shown that Nav1.3 is an important factor in the development of chronic pain in the response to nerve injury [61]. In our studies, we did not observe upregulation of Nav1.3 transcript (*Scn3a*) in DRG L5, although it was mainly upregulated in studies on more robust models of neuropathic pain caused by nerve injury [61, 62]. Interestingly, we observed upregulation of *Scn3a* in lumbar SC that may reflect sensitization of central pain pathways, as it was observed previously [63]. These results demonstrate that development of OA rats can trigger changes in sodium channel expression and suggest a functional link between Nav1.3 expression and neuronal firing associated with neuropathic pain.

## Conclusions

The present study showed the characteristics and the time course of pain behavior in a MIA-induced rodent knee OA model during 28 days after injection, both in the aspect of local hypersensitivity and developing over time signs of neuropathy. Acknowledgment of the presence of neuropathy component in OA-related pain should lead to changes in patient’s treatment paradigms, especially that our behavioral data corresponded nicely with subchondral bone alterations measured by the means of microtomography.

The main result of our studies is characterization of molecular changes present in DRG L5 and SC during progression of OA. Our experiments revealed upregulation of key factors involved in neuropathic pain development in an animal model of OA. Greatest alterations were observed for growth factors, peptidergic signaling molecules, and voltage dependent sodium channels—all indicating the neural origin of the neuropathic component of OA-related pain and declining the involvement of inflammation in that process. Especially that changes in the expression of proinflammatory cytokines in examined tissues were relatively of low significance. These results suggest that persistent cartilage degeneration within the OA joint leads to molecular changes within DRG L5 and lumbar SC that correspond to functional changes observed in neuropathic pain conditions.

However, one must note the limitations of the study. Observed molecular changes on the tissue level of DRG and SC were not cell-type specific. While qRT-PCR was performed on homogenized nervous tissue containing both neurons and glia cells, IHC was also lacking double-staining with cell-specific markers. Thus, the question about whether these molecular changes affect strictly neuronal cell or is rather mediated by glial cells, remains to be open. Furthermore, the current study focused exclusively on a subset of the factors that exhibited alterations in the DRGs and spinal cord of OA-afflicted animals, specifically IL1β and CGRP. This deliberate narrowing of scope underscores the necessity for subsequent investigations to reach into the remaining factors. Consequently, there is a strong need for further studies to substantiate the presence of a neuropathic pain component within the context of OA.

Our data bring better understanding of mechanism behind difficult to treat chronic OA pain and support the hypothesis of neuropathic-like component in the MIA model. This might serve as compelling evidence for development of novel therapeutic agents for OA treatment, although functional coupling to pain development remains yet to be shown.

### Supplementary Information

Below is the link to the electronic supplementary material.

Supplementary file1 (PPTX 1931 KB)

Supplementary file2 (PDF 37 KB)

Supplementary file3 (PDF 45 KB)

## Data Availability

The datasets generated during and/or analyzed during the current study are not publicly available but are available from the corresponding author on reasonable request.

## References

[1] Cross M, Smith E, Hoy D (2014). The global burden of hip and knee osteoarthritis: estimates from the Global Burden of Disease 2010 study. Ann Rheum Dis.

[2] Hochman JR, French MR, Bermingham SL, Hawker GA (2010). The nerve of osteoarthritis pain. Arthritis Care Res.

[3] Kosek E, Ordeberg G (2000). Abnormalities of somatosensory perception in patients with painful osteoarthritis normalize following successful treatment. Eur J Pain.

[4] Dimitroulas T, Duarte RV, Behura A, Kitas GD, Raphael JH (2014). Neuropathic pain in osteoarthritis: a review of pathophysiological mechanisms and implications for treatment. Semin Arthritis Rheum.

[5] Felson DT (2009). Developments in the clinical understanding of osteoarthritis. Arthritis Res Ther.

[6] Phillips K, Clauw DJ (2013). Central pain mechanisms in the rheumatic diseases: future directions. Arthritis Rheum.

[7] Kidd BL (2006). Osteoarthritis and joint pain. Pain.

[8] Schaible HG, Richter F, Ebersberger A (2009). Joint pain. Exp Brain Res.

[9] Schuelert N, Zhang C, Mogg AJ (2010). Paradoxical effects of the cannabinoid CB2 receptor agonist GW405833 on rat osteoarthritic knee joint pain. Osteoarthritis Cartilage.

[10] Miller RE, Kim YS, Tran PB (2018). Visualization of peripheral neuron sensitization in a surgical mouse model of osteoarthritis by in vivo calcium imaging. Arthritis Rheumatol.

[11] Neogi T, Guermazi A, Roemer F (2016). Association of joint inflammation with pain sensitization in knee osteoarthritis: the Multicenter Osteoarthritis Study. Arthritis Rheumatol.

[12] Orita S, Ishikawa T, Miyagi M, et al (2011) Pain-related sensory innervation in monoiodoacetate-induced osteoarthritis in rat knees that gradually develops neuronal injury in addition to inflammatory pain. BMC Musculoskelet Disord. 12(1). 10.1186/1471-2474-12-134 10.1186/1471-2474-12-134 PMC3142251 21679434

[13] Arendt-Nielsen L, Nie H, Laursen MB (2010). Sensitization in patients with painful knee osteoarthritis. Pain.

[14] Liu W, Li C, Tan FCK, et al (2020) Cerebrospinal fluid of chronic osteoarthritic patients induced interleukin-6 release in human glial cell-line T98G. *BMC Anesthesiol*. 20(1). 10.1186/S12871-020-00985-0 10.1186/s12871-020-00985-0 PMC7093964 32213162

[15] Thakur M, Rahman W, Hobbs C, Dickenson AH, Bennett DLH (2012) Characterisation of a peripheral neuropathic component of the rat monoiodoacetate model of osteoarthritis. PloS One. 7(3). 10.1371/JOURNAL.PONE.0033730 10.1371/journal.pone.0033730 PMC3312347 22470467

[16] Graven-Nielsen T, Arendt-Nielsen L (2002). Peripheral and central sensitization in musculoskeletal pain disorders: an experimental approach. Curr Rheumatol Rep.

[17] Guingamp C, Gegout-Pottie P, Philippe L, Terlain B, Netter P, Gillet P (1997). Mono-iodoacetate-induced experimental osteoarthritis: a dose-response study of loss of mobility, morphology, and biochemistry. Arthritis Rheum.

[18] Malek N, Mrugala M, Makuch W (2015). A multi-target approach for pain treatment: dual inhibition of fatty acid amide hydrolase and TRPV1 in a rat model of osteoarthritis. Pain.

[19] Lolignier S, Eijkelkamp N, Wood JN (2015). Mechanical allodynia. Pflugers Arch.

[20] Limaye A (2012) Drishti: a volume exploration and presentation tool. In: *Developments in X-Ray Tomography VIII*. Vol 8506. SPIE; 85060X. 10.1117/12.935640

[21] Schneider CA, Rasband WS, Eliceiri KW (2012). NIH Image to ImageJ: 25 years of image analysis. Nat Methods.

[22] Skyscan N V (1987) Structural parameters measured by the Skyscan ^TM^ CT-analyser software . *Bone*. m:1–15.

[23] Nitzan-Luques A, Minert A, Devor M, Tal M (2013). Dynamic genotype-selective “phenotypic switching” of CGRP expression contributes to differential neuropathic pain phenotype. Exp Neurol.

[24] Bryk M, Chwastek J, Mlost J, Kostrzewa M, Starowicz K (2021). Sodium monoiodoacetate dose-dependent changes in matrix metalloproteinases and inflammatory components as prognostic factors for the progression of osteoarthritis. Front Pharmacol.

[25] Lee JH, Chun KJ, Kim HS (2012). Alteration patterns of trabecular bone microarchitectural characteristics induced by osteoarthritis over time. Clin Interv Aging.

[26] Boakye PA, Tang S, Smith PA (2021). Mediators of neuropathic pain; focus on spinal microglia, CSF-1, BDNF, CCL21, TNF-α, Wnt ligands, and interleukin 1β. Front Pain Res.

[27] Ruscheweyh R, Forsthuber L, Schoffnegger D, Sandkühler J (2007). Modification of classical neurochemical markers in identified primary afferent neurons with Aβ-, Aδ-, and C-fibers after chronic constriction injury in mice. J Comp Neurol.

[28] Stemkowski PL, Garcia-Caballero A, Gadotti VM (2017). Identification of interleukin-1 beta as a key mediator in the upregulation of Cav3.2–USP5 interactions in the pain pathway. Mol Pain.

[29] Aso K, Izumi M, Sugimura N, Okanoue Y, Ushida T, Ikeuchi M (2016). Nociceptive phenotype alterations of dorsal root ganglia neurons innervating the subchondral bone in osteoarthritic rat knee joints. Osteoarthritis Cartilage.

[30] Zelenka M, Schafers M, Sommer C (2005). Intraneural injection of interleukin-1beta and tumor necrosis factor-alpha into rat sciatic nerve at physiological doses induces signs of neuropathic pain. Pain.

[31] Binshtok AM, Wang H, Zimmermann K (2008). Nociceptors are interleukin-1beta sensors. J Neurosci.

[32] Bai Y, Yang Q, Chen P, Wang X (2023). Repetitive transcranial magnetic stimulation regulates neuroinflammation in neuropathic pain. Front Immunol.

[33] Schaible HG, Segond von Banchet G, Boettger MK (2010). The role of proinflammatory cytokines in the generation and maintenance of joint pain. Ann N Y Acad Sci.

[34] Driscoll C, Chanalaris A, Knights C (2016). Nociceptive sensitizers are regulated in damaged joint tissues, including articular cartilage, when osteoarthritic mice display pain behavior. Arthritis Rheumatol.

[35] Richner M, Ulrichsen M, Elmegaard SL, Dieu R, Pallesen LT, Vaegter CB (2014). Peripheral nerve injury modulates neurotrophin signaling in the peripheral and central nervous system. Mol Neurobiol.

[36] Huang ZJ, Song XJ (2008) Differing alterations of sodium currents in small dorsal root ganglion neurons after ganglion compression and peripheral nerve injury. *Mol Pain*. 4. 10.1186/1744-8069-4-20 10.1186/1744-8069-4-20 PMC2427019 18513405

[37] Cho HJ, Kim JK, Park HC (1998). Changes in brain-derived neurotrophic factor immunoreactivity in rat dorsal root ganglia, spinal cord, and gracile nuclei following cut or crush injuries. Exp Neurol.

[38] Mannion RJ, Costigan M, Decosterd I (1999). Neurotrophins: peripherally and centrally acting modulators of tactile stimulus-induced inflammatory pain hypersensitivity. Proc Natl Acad Sci.

[39] Fukuoka T, Kondo E, Dai Y, Hashimoto N, Noguchi K (2001). Brain-derived neurotrophic factor increases in the uninjured dorsal root ganglion neurons in selective spinal nerve ligation model. J Neurosci Off J Soc Neurosci.

[40] Cappoli N, Tabolacci E, Aceto P, Dello Russo C (2020). The emerging role of the BDNF-TrkB signaling pathway in the modulation of pain perception. J Neuroimmunol..

[41] Grau JW, Huang YJ, Turtle JD (2017). When pain hurts: nociceptive stimulation induces a state of maladaptive plasticity and impairs recovery after spinal cord injury. J Neurotrauma.

[42] Seybold VS (2009). The role of peptides in central sensitization. Handb Exp Pharmacol.

[43] Schaible HG, Freudenberger U, Neugebauer V, Stiller RU (1994). Intraspinal release of immunoreactive calcitonin gene-related peptide during development of inflammation in the joint in vivo—a study with antibody microprobes in cat and rat. Neuroscience.

[44] Donaldson LF, Harmar AJ, McQueen DS, Seckl JR (1992). Increased expression of preprotachykinin, calcitonin gene-related peptide, but not vasoactive intestinal peptide messenger RNA in dorsal root ganglia during the development of adjuvant monoarthritis in the rat. Brain Res Mol Brain Res.

[45] Oku R, Satoh M, Fujii N, Otaka A, Yajima H, Takagi H (1987). Calcitonin gene-related peptide promotes mechanical nociception by potentiating release of substance P from the spinal dorsal horn in rats. Brain Res.

[46] Sun RQ, Lawand NB, Willis WD (2003). The role of calcitonin gene-related peptide (CGRP) in the generation and maintenance of mechanical allodynia and hyperalgesia in rats after intradermal injection of capsaicin. Pain.

[47] Bird GC, Han JS, Fu Y, Adwanikar H, Willis WD, Neugebauer V (2006). Pain-related synaptic plasticity in spinal dorsal horn neurons: role of CGRP. Mol Pain.

[48] Adwanikar H, Ji G, Li W, Doods H, Willis WD, Neugebauer V (2007). Spinal CGRP1 receptors contribute to supraspinally organized pain behavior and pain-related sensitization of amygdala neurons. Pain.

[49] Dong T, Chang H, Zhang F (2015). Calcitonin gene-related peptide can be selected as a predictive biomarker on progression and prognosis of knee osteoarthritis. Int Orthop.

[50] Lindh C, Liu Z, Welin M, Ordeberg G, Nyberg F (1999). Low calcitonin gene-related, peptide-like immunoreactivity in cerebrospinal fluid from chronic pain patients. Neuropeptides.

[51] Noguchi K, de León M, Nahin RL, Senba E, Ruda MA (1993). Quantification of axotomy-induced alteration of neuropeptide mRNAs in dorsal root ganglion neurons with special reference to neuropeptide Y mRNA and the effects of neonatal capsaicin treatment. J Neurosci Res.

[52] Shi TJ, Cui JG, Meyerson BA, Linderoth B, Hökfelt T (1999). Regulation of galanin and neuropeptide Y in dorsal root ganglia and dorsal horn in rat mononeuropathic models: possible relation to tactile hypersensitivity. Neuroscience.

[53] Benoliel R, Eliav E, Iadarola MJ (2001). Neuropeptide Y in trigeminal ganglion following chronic constriction injury of the rat infraorbital nerve: is there correlation to somatosensory parameters?. Pain.

[54] Brumovsky PR, Bergman E, Liu HX, Hökfelt T, Villar MJ (2004). Effect of a graded single constriction of the rat sciatic nerve on pain behavior and expression of immunoreactive NPY and NPY Y1 receptor in DRG neurons and spinal cord. Brain Res.

[55] Solway B, Bose SC, Corder G, Donahue RR, Taylor BK (2011). Tonic inhibition of chronic pain by neuropeptide Y. Proc Natl Acad Sci U S A.

[56] Gutierrez S, Alvarado-Vázquez PA, Eisenach JC, Romero-Sandoval EA, Boada MD (2019). Tachykinins modulate nociceptive responsiveness and sensitization: in vivo electrical characterization of primary sensory neurons in tachykinin knockout (Tac1 KO) mice. Mol Pain.

[57] Pantaleo N, Chadwick W, Park SS (2012). The mammalian tachykinin ligand-receptor system: an emerging target for central neurological disorders. CNS Neurol Disord - Drug Targets.

[58] Devor M, Govrin-Lippmann R, Angelides K (1993). Na+ channel immunolocalization in peripheral mammalian axons and changes following nerve injury and neuroma formation. J Neurosci.

[59] Dib-Hajj SD, Rush AM, Cummins TR (2005). Gain-of-function mutation in Na v 1.7 in familial erythromelalgia induces bursting of sensory neurons. Brain..

[60] Li Y, North RY, Rhines LD (2018). DRG voltage-gated sodium channel 1.7 is upregulated in paclitaxel-induced neuropathy in rats and in humans with neuropathic pain. J Neurosci Off J Soc Neurosci..

[61] Chen HP, Zhou W, Kang LM (2014). Intrathecal miR-96 inhibits Nav1.3 expression and alleviates neuropathic pain in rat following chronic construction injury. Neurochem Res..

[62] Su S, Shao J, Zhao Q, et al (2017) MiR-30b attenuates neuropathic pain by regulating voltage-gated sodium channel Nav1.3 in rats. Front Mol Neurosci. 10. 10.3389/FNMOL.2017.00126 10.3389/fnmol.2017.00126 PMC5418349 28529474

[63] Hains BC, Klein JP, Saab CY, Craner MJ, Black JA, Waxman SG (2003). Upregulation of sodium channel Nav1.3 and functional involvement in neuronal hyperexcitability associated with central neuropathic pain after spinal cord injury. J Neurosci Off J Soc Neurosci..

