# Supplementary material for: Description of Novel Molecular Factors in Lumbar DRGs and Spinal Cord Factors Underlying Development of Neuropathic Pain Component in the Animal Model of Osteoarthritis
Source: Mol Neurobiol. 2023 Sep 21;61(3):1580–92. doi: 10.1007/s12035-023-03619-x (PMC10896862; doi:10.1007/s12035-023-03619-x)
Supplement: Supplementary file 1 — Supplementary file1 (PPTX 1931 KB) [file 12035_2023_3619_MOESM1_ESM.pptx]

## Slide 1
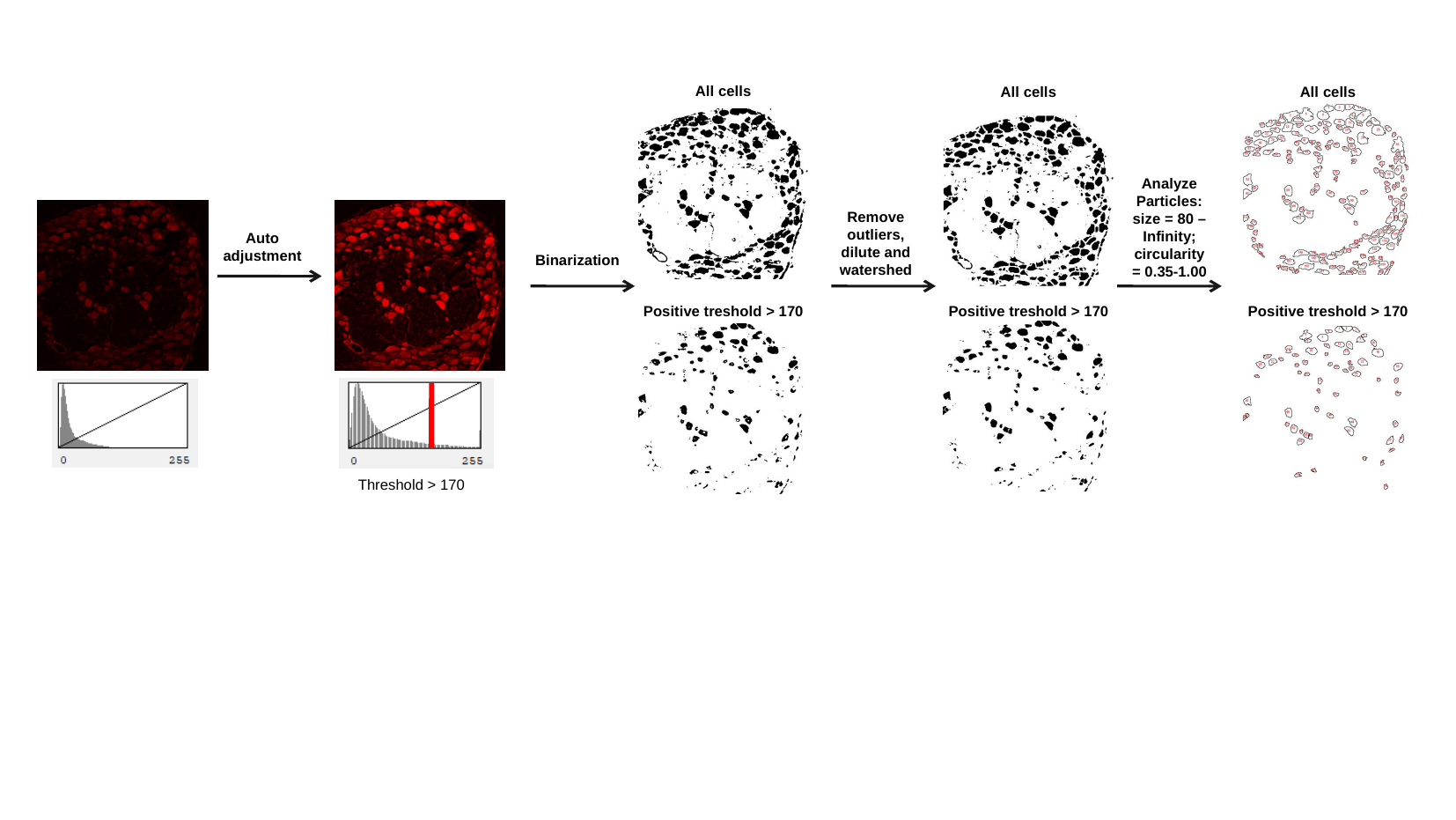

All cells
All cells
All cells
Analyze Particles: size = 80 – Infinity; circularity = 0.35-1.00
Remove outliers, dilute and watershed
Auto
adjustment
Binarization
Positive treshold > 170
Positive treshold > 170
Positive treshold > 170
Threshold > 170
