# Supplementary figures and images for: Description of Novel Molecular Factors in Lumbar DRGs and Spinal Cord Factors Underlying Development of Neuropathic Pain Component in the Animal Model of Osteoarthritis
Source: Mol Neurobiol. 2023 Sep 21;61(3):1580–92. doi: 10.1007/s12035-023-03619-x (PMC10896862; doi:10.1007/s12035-023-03619-x)

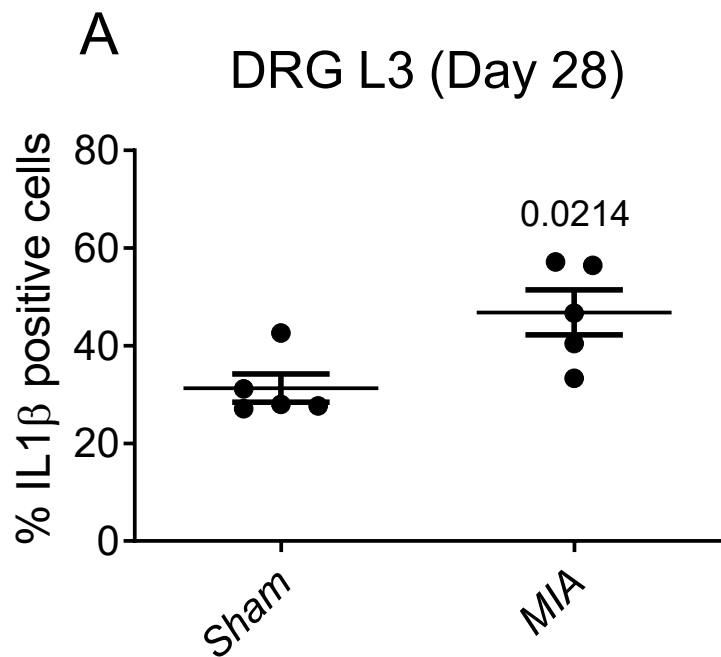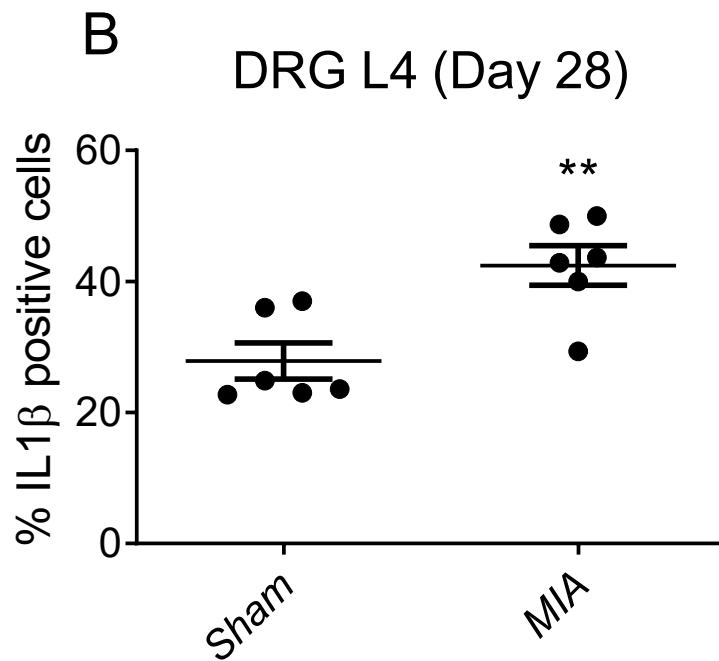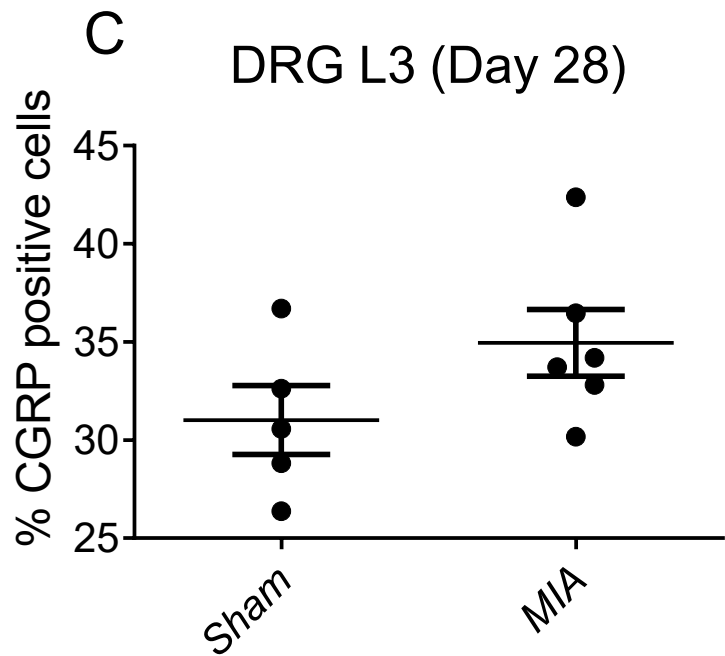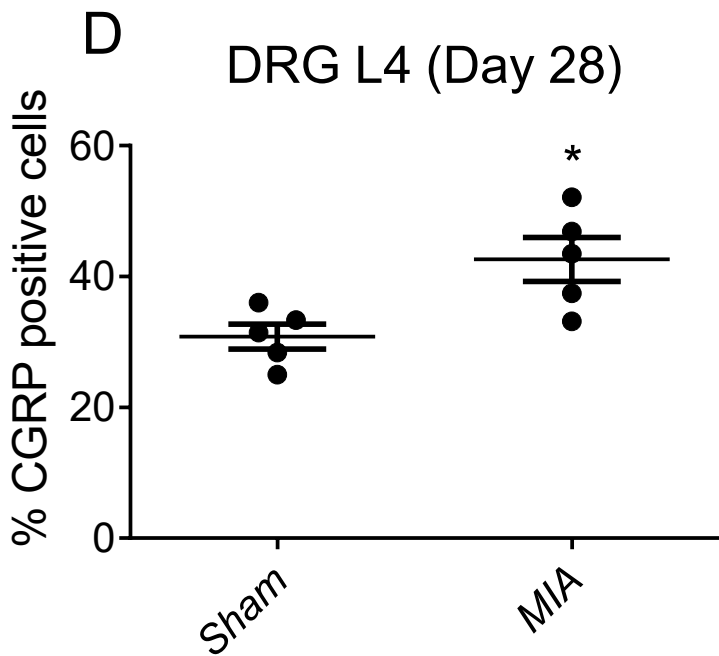

Supplement: Supplementary file 2 — Supplementary file2 (PDF 37 KB) [file 12035_2023_3619_MOESM2_ESM.pdf]

## Dorsal root ganglion L3

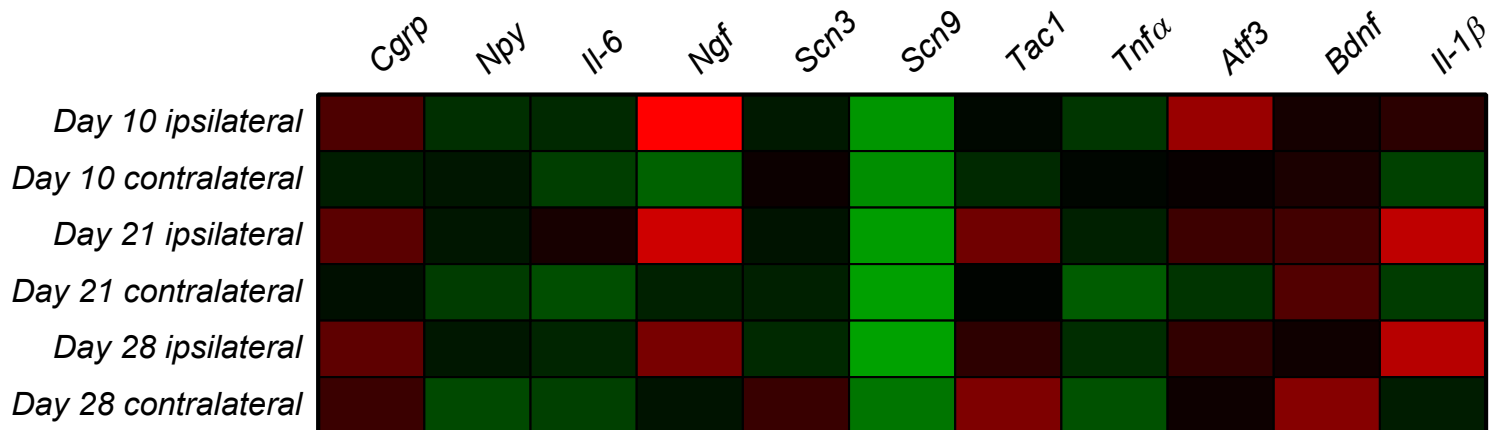

## Dorsal root ganglion L4

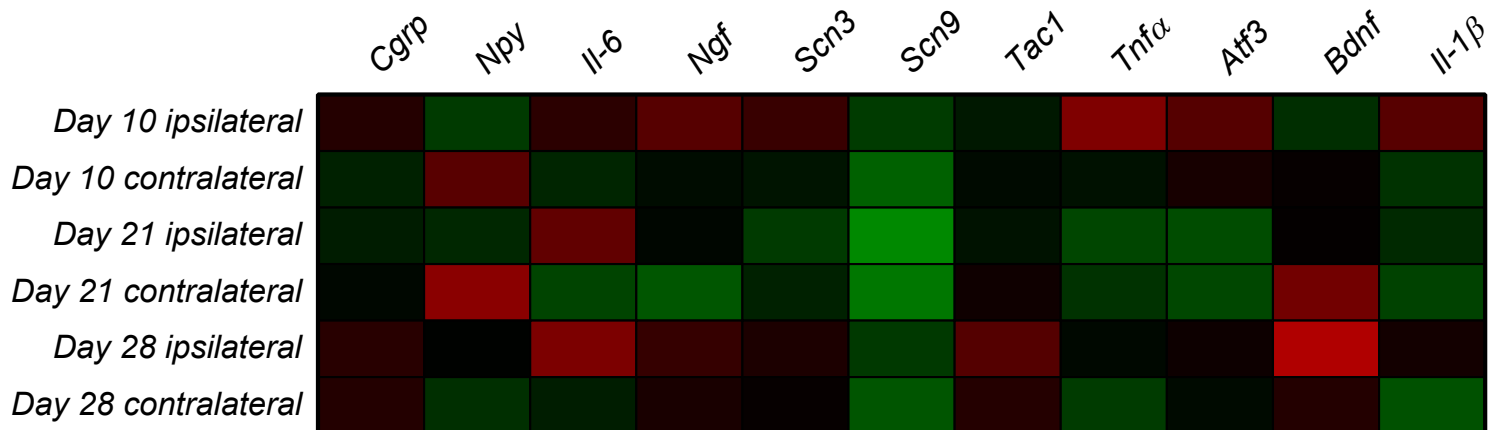

Relative expression change (%)

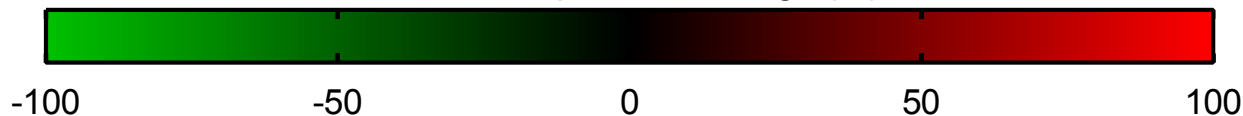

Supplement: Supplementary file 3 — Supplementary file3 (PDF 45 KB) [file 12035_2023_3619_MOESM3_ESM.pdf]
